# Plant Adaptability and Vegetation Differentiation in the Coastal Beaches of Yellow–Bohai Sea in China

**DOI:** 10.3390/ijerph19042225

**Published:** 2022-02-16

**Authors:** Qian Dong, Qingqing Zhang, Anbang Liao, Chi Xu, Maosong Liu

**Affiliations:** School of Life Sciences, Nanjing University, Nanjing 210046, China; njuskydq@smail.nju.edu.cn (Q.D.); qingqingzh2013@126.com (Q.Z.); mg20300065@smail.nju.edu.cn (A.L.); xuchi@nju.edu.cn (C.X.)

**Keywords:** community–soil corresponding relationship, ecological strategy, vegetation differentiation, coastal wetlands ecosystem, *Spartina alterniflora*, *Suaeda salsa*, *Phragmites australis*

## Abstract

To identify the key soil factors influencing the vegetation differentiation in the coastal tidal flats of the Yellow−Bohai Sea in China, this study investigated the corresponding relationship between the *Spartina alterniflora* (SA), *Suaeda salsa* (SS), and *Phragmites australis* (PA) communities and their respective soil factors with published data, and combined the ecological strategy for analysis. The results showed a corresponding relationship between community and soil factors. The SA community had a lower bulk density (BD) and higher soil total nitrogen (TN), and the SS community was the opposite, while the PA community had the lowest salinity and higher TN. BD, salinity and TN acted as the main soil factors driving vegetation differentiation, but the explained proportion of the three factors to vegetation differentiation changed by season and region. Considering that higher TN facilitates the competitors, salinity represents the environmental stresses, and BD is positively related to the frequency of perturbation in the specific habitat in the study area, SA, SS and PA could be recognized as C–S, S–R and C strategic species to some extent. It is likely that some coexistent mechanisms for invasive and local species will be developed, especially the SS community which seriously shrunk recently but served as an important habitat for waterfowls in tidal flat habitats.

## 1. Introduction

Since *Spartina alterniflora* (SA) was introduced to China in the 1970s, the vegetation of tidal flats by the Yellow−Bohai Sea (YBS) in China has been changed. The expansion of the SA community (SAc) led to the shrinkage of some local vegetation [1,2], and changed succession processes [1] and species habitats [3].

With the consistent expansion, *Suaeda salsa* (SS), *Phragmites australis* (PA) and SA have become the three dominant species now in the coastal beaches of YBS, which normally existed as monodominant communities or mosaic communities. Soil physical and chemical features could affect the distribution pattern of communities [4], while community succession could change site conditions in the meantime [5], but domination of the vegetation differentiation was conditional. It might be hydrological conditions, soil physical properties [6,7], or nutrient conditions [8,9], or something else. Some studies found it possible to affect the direction of community succession by changing soil factors [10]. Revealing the driving factors for vegetation differentiation might help the restoration of wetland ecosystems.

Usually, soil factors change with season, and the variations were different among the factors to some extent. Just like the study in the tidal flats in the estuary of the Yellow River, the soil NO_3_^−^−N, NH_4_^+^−N, and soil organic carbon content (SOC) in the SS and PA communities (SSc, PAc) was lower in summer than that in spring and autumn, but soil water content was lower in spring than that in summer and autumn [11]. Soil bulk density (BD) was significantly lower in spring than that in summer and autumn, while soil salinity was usually higher in spring than that in summer or autumn [12]. It is necessary to identify the function of soil factors in vegetation differentiation by season and region.

In this paper, based on the published materials, we investigated the corresponding relationship between communities and soil factors, to identify:

1. The roles of the main soil factors in vegetation differentiation, and their regional and seasonal differences; and
2. Key species’ strategic features and the mechanism of vegetation differentiation.

## 2. Materials and Methods

### 2.1. Study Area

**Figure 1:**
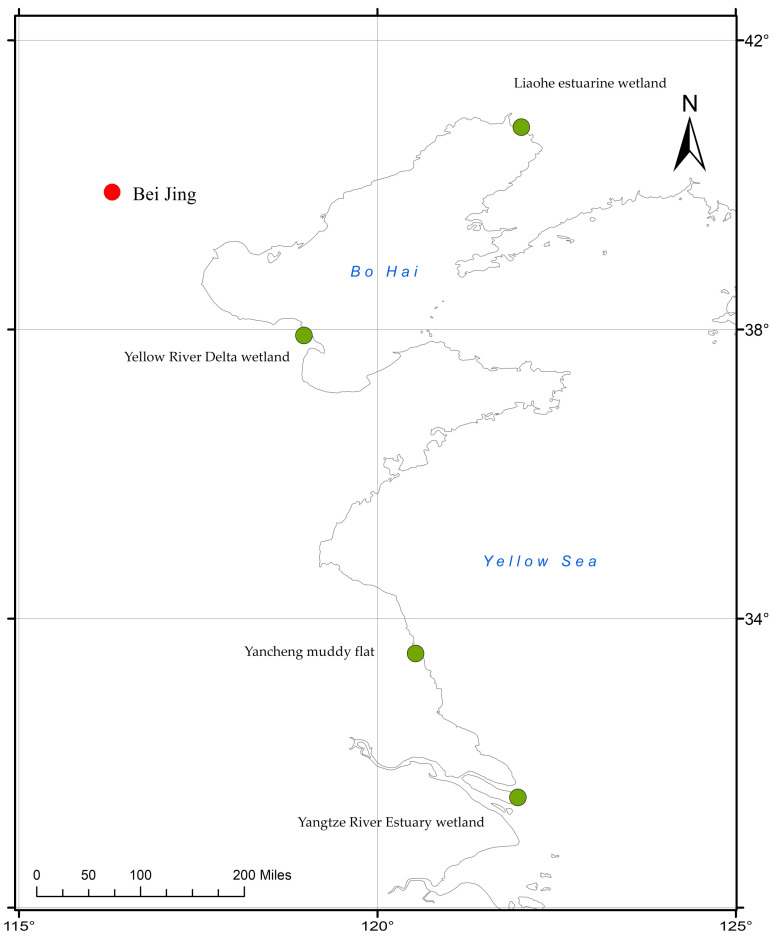
Map of study area. Green points represent four main study areas.

The tidal flats of YBS are mainly distributed between 30° N and 42° N, with a total area of about 182.33 × 10^4^ hm^2^, accounting for 83.24% of the total area of coastal wetland in China (Figure 1), where SAc, SSc, and PAc dominate the saltmarsh vegetation. Intertidal mudflats play an irreplaceable role for the migration and breeding of waterbirds around the world [13,14], maintaining shorebird diversity [15].

The temperature and precipitation in the coastal beaches of YBS in China show evident latitudinal variation. It can be roughly divided into northern and southern areas according to 34°N, which is the approximate latitude of the 0 ℃ isotherm in China in January. The northern area by the Bohai Sea, including Liaohe estuarine wetland (41.07° N, 122.00° E) and Yellow River Delta wetland (37.92° N, 118.98° E), is mainly affected by the temperate monsoon climate. The annual precipitation in the north is 400–800 mm, which is mainly concentrated in July and August. The average evaporation is also about 1024 mm per year.

The climate type in the southern area by the Yellow Sea includes the Yancheng muddy flat (33.52° N, 120.37° E) and the Yangtze River estuary wetland (31.53° N, 121.96° E), which has a subtropical monsoon climate. The annual average precipitation is more than 800 mm, which changes significantly every month and is generally concentrated in summer. The average annual evaporation is 931 mm.

### 2.2. Statistical Analysis

EndNote and NoteExpress were used to search relevant studies in ScienceDirect, Web of Science, and China National Knowledge Infrastructure (CNKI) databases with the keywords of “*Spartina alterniflora*”, “*Suaeda salsa*”, “*Phragmites australis*”, “soil” and “tidal flat”. The included experimental samples must have met the following conditions:

- The study area is limited to the tidal flat of the YBS in China from 30°–42° N;
- The exact location of the study site and the time of sampling (at least specific to season) should be specified in the paper; and
- At least one type of data of soil physical and chemical properties should be included, such as soil bulk density (BD), soil salinity, total organic carbon (TOC), total nitrogen (TN), total phosphorus (TP), pH, and so forth, which correspond to the three typical mono-dominant communities.

For the data published in graphs, the numerical calculation was performed by GetData (2.26). As a result, a total of 64 articles were selected (Table A1), and five factors of BD, salinity, pH, TN and TP were selected due to data availability.

According to the local plant phenology, the seasons were divided as spring (March to May), summer (June to August), autumn (September to November) and winter (December to February). Statistical analyses were carried out with Origin 2022 to generate boxplots. Differences of soil factors corresponding to the communities in different regions or seasons were calculated using analysis of variance (ANOVA), and a LSD post hoc test was used with SPSS.

Considering the overlap of both numerical ranges and probabilities, the probability density curve overlap was used to calculate the overlap of the soil factors’ variation range of the communities by R packages ‘overlapping’. According to the theory of ecological niches, an overlap over 0.3 means that the overlap makes sense. If the figure is over 0.6, the overlap is significant [16].

The contribution and correlation of soil factors to vegetation differentiation in different regions and seasons were calculated with redundancy analysis (RDA). To eliminate the arch effect, according to detrended correspondence analysis (DCA) results, the axis lengths of gradients were less than 3.0, and the RDA method was selected for analysis. The RDA graph and the statistical test of RDA based on ANOVA was completed by using RStudio 4.0.5. The simple effect was equal to the explained proportion of constrained ordination with only a single soil factor. After the data were normalized to the range of 0 to 1, ternary plots were made according to the C–S–R model [17]. Graphs were made with the R package ‘ggtern’.

## 3. Results

### 3.1. Characteristics and Variation of Soil Factors in Typical Communities

There are differences in soil factors corresponding to communities. Among the three typical communities, the differences in soil BD (*p* = 0.000), salinity (*p* = 0.000) and TN (*p* = 0.000) were significant, while the differences in soil pH (*p* = 0.670) and TP (*p* = 0.701) were not significant.

**Figure 2:**
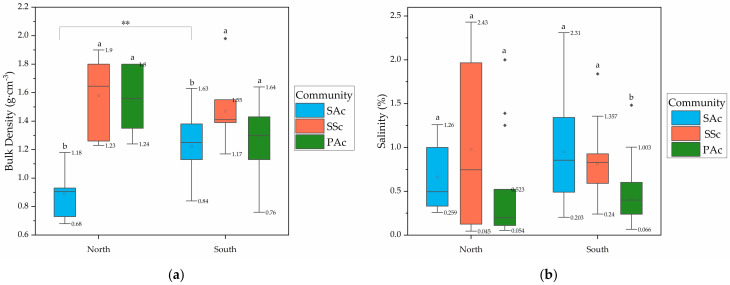

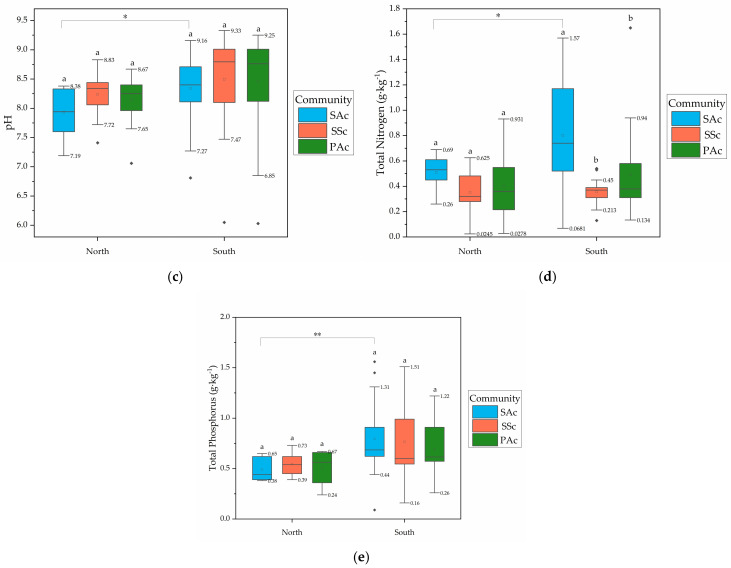
Box plots of the differences in soil factors corresponding to the three communities by region: (**a**) bulk density; (**b**) salinity; (**c**) pH; (**d**) TN; and (**e**) TP. Different colors represent different communities. Boxes show the 25th and 75th percentiles and medians (thick lines), while staples indicate the smallest and highest values (excluding outliers). Outliers are shown as solid circles. The range of whiskers is marked in the figure. Lowercase letters indicate the significance of differences between communities within a group, where “a, b” indicates significant difference among the three communities (*p* < 0.05). Differences between groups are marked with an asterisk in the figure, and are not marked if the difference is not significant, where ‘**’ represents *p* < 0.01, ‘*’ represents *p* < 0.05.

The relative significance of the differences varies with season and region. Regionally, soil BD and salinity in the SAc were lower in the north than those in the south, while in PAc and SSc they were higher in the north. The soil pH, TN and TP were lower in the north in all three communities (Figure 2). This may be because the precipitation in the north is less than that in the south, the dry−wet alternating frequency of soil is low, and the SAc is relatively dense, which has a certain role in blocking water flow, resulting in high soil water content, high soil porosity and low salt content in the north. However, the situation is different in SSc and PAc. Due to the large evaporation in the north, in SSc and PAc with less dense vegetation, it will lead to soil water loss, soil compaction, increased salinity, and the loss of nutrient elements in the soil, such as nitrogen and phosphorus.

**Figure 3:**
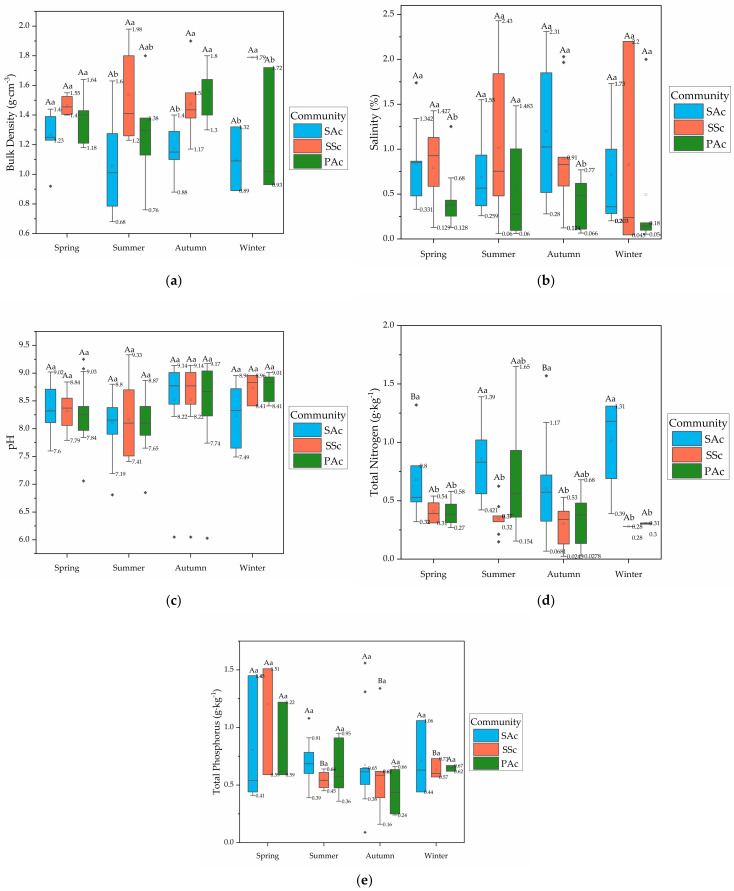
Box plots of the differences in soil factors corresponding to the three communities by season: (**a**) bulk density; (**b**) salinity; (**c**) pH; (**d**) TN; and (**e**) TP. Different colors represent different communities. Boxes show the 25th and 75th percentiles and medians (thick lines), while staples indicate the smallest and highest values (excluding outliers). Outliers are shown as solid circles. The range of whiskers is marked in the figure. Lowercase letters indicate the significance of differences between communities in the same season, where “a, b” indicates significant difference among the three communities (*p* < 0.05) Capital letters indicate the significance of differences between seasons in each community, where “A, B” indicates significant difference among the four seasons (*p* < 0.05).

Seasonally, soil pH and TP have similar seasonal variation characteristics among communities. Soil pH in autumn was higher than that in spring and the lowest in summer, and soil TP in autumn and winter was higher than that in spring and summer. However, the variation characteristics of BD, salinity and TN among seasons are different in different communities. For example, BD was the highest in spring in SAc, while in SSc it was the highest in summer, and in PAc it was the highest in autumn (Figure 3). This may be related to the difference of plant life history and nutrient utilization.

Among communities, soil BD was the highest in SSc, followed by PAc and SAc as a whole, while the salinity was highest in SAc, followed by SSc and PAc. The soil pH of SSc was slightly higher than that of PAc, and of SAc was the lowest. The content was the highest for both TN and TP in SAc, but TN of PAc was higher than that SSc, while TP of PAc was lower than that of SSc (Figure 2 and Figure 3). Both SA and PA have complex roots, which need soil with large porosity to extend their roots. SS is an annual plant with shallow roots, which can be planted in soil with high BD. Both SA and SS can tolerate high salt stress, but PA cannot. SA is a C4 plant, which is suitable for colonization in high-nutrient soil.

Comparing the variation range and the overlap of soil factors among communities, BD significantly overlapped between PAc and SSc in the north, salinity of SAc and SSc overlapped considerably in the south, while salinity of PAc overlapped less with the other two communities as a whole. The range of TN and TP in the three communities was overlapped less in winter, and the overlap of TP among the three communities was small in autumn (Table 1). Generally speaking, the selection of soil environment by the three communities overlaps to a certain extent. The three communities always coexist in the same tidal flat in the form of mosaic community, and the habitat heterogeneity is relatively small. However, we could still recognize BD and salinity as identifiers of the three communities due to the small overlap, respectively.

### 3.2. Redundancy Analysis between Soil Factors and Communities

**Figure 4:**
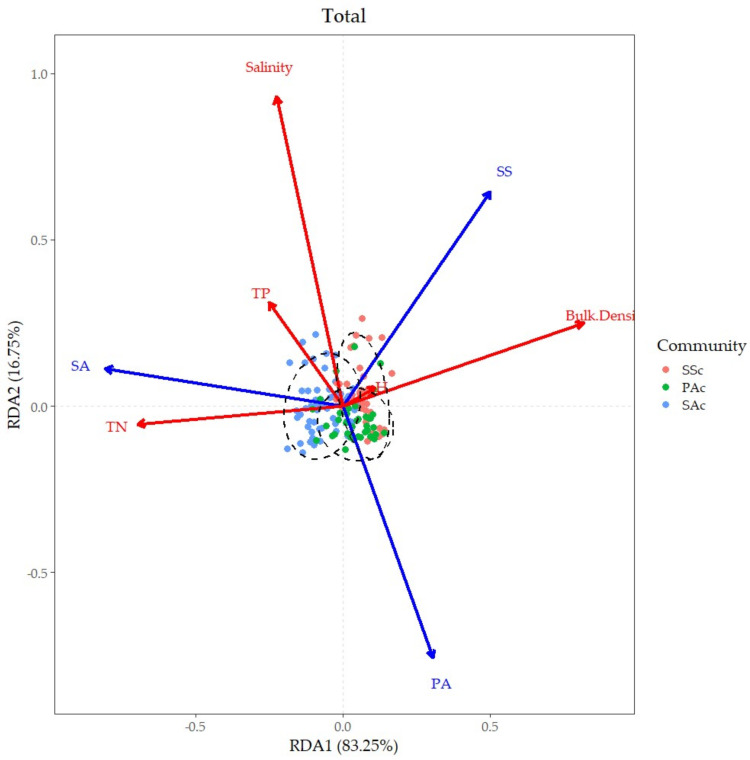
A two-dimensional graph of RDA ordination for communities and soil factors. The cumulative interpretation rates of the two sorting axes are labeled on the axis labels. Different colors represent different communities. The red axes represent the soil factors. The blue axes represent the species. The solid dots represent the soil factors corresponding to the communities.

RDA was used to explore the ecological driving factors on vegetation differentiation, and the statistical results are shown in Table 2 and Table 3. The results show that TN has a significant positive correlation with SAc, BD and salinity were always positively related to SSc, and there exists a negative correlation between salinity and the distribution of PAc (Figure 4). This may reveal the indicative relationship between the three communities and the corresponding environmental factors.

**Figure A1:**
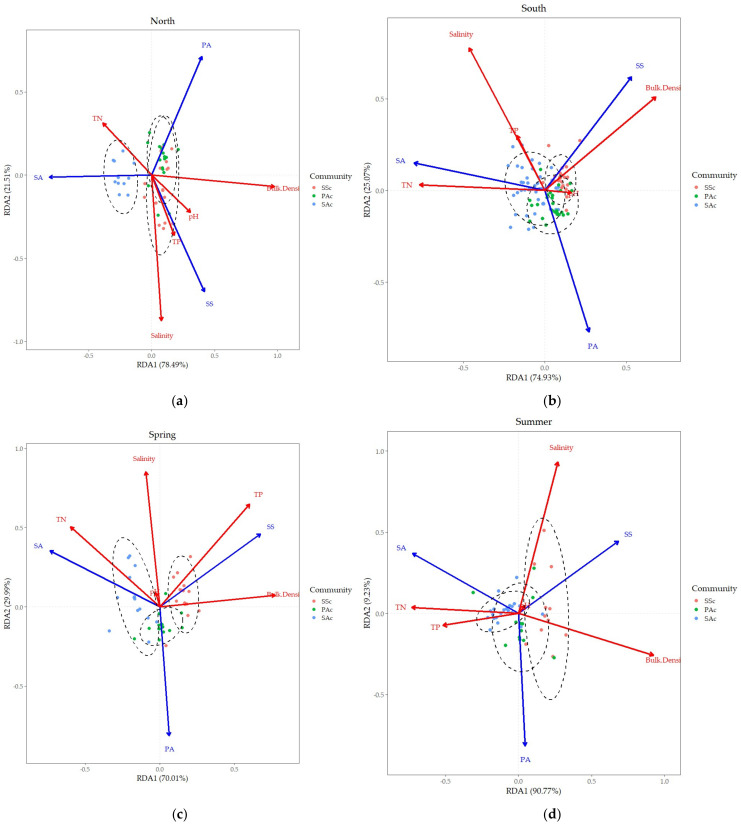

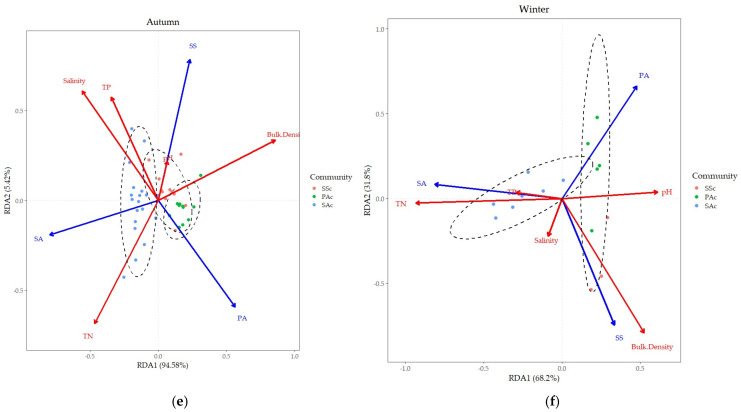
A two-dimensional graph of RDA ordination for communities and soil factors in different regions and seasons: (**a**) north; (**b**) south; (**c**) spring; (**d**) summer; (**e**) autumn; and (**f**) winter. The cumulative interpretation rates of the two sorting axes are labeled on the axis labels. Different colors represent different communities. The red axes represent the soil factors. The blue axes represent the species. The solid dots represent the soil factors corresponding to the communities.

In contrast, although the corresponding relationship and the distribution trend of axis was similar, the separation degree of the three communities was different in the north and south and the four seasons (Figure A1, and results of the statistical test in Table A2 and Table A3). For example, SAc separated significantly with the other two communities in the north and in the winter.

Comparing the explained proportion of each soil factor, there were a few differences in the ranking of factors on vegetation differentiation in the north and south, but the explained proportion was different (Table 4). The simple effect of BD in the north was dramatically high, while TN and salinity explained more proportion in the south.

Seasonally, the differences of ranking and explained proportion were more significant. BD and TN played important roles throughout the whole year. However, salinity seemed to contribute the most in autumn. The simple effect of community types by TP ranked higher in spring and summer than in other seasons.

### 3.3. Distribution of Three Communities along Soil Factor Gradient

According to the above results, BD, salinity and TN had a relatively obvious effect on vegetation differentiation. Therefore, ternary plots of the three soil factors by region and season were made to reveal the main distribution of the three communities based on Grime’s C−S−R model.

**Figure 5:**
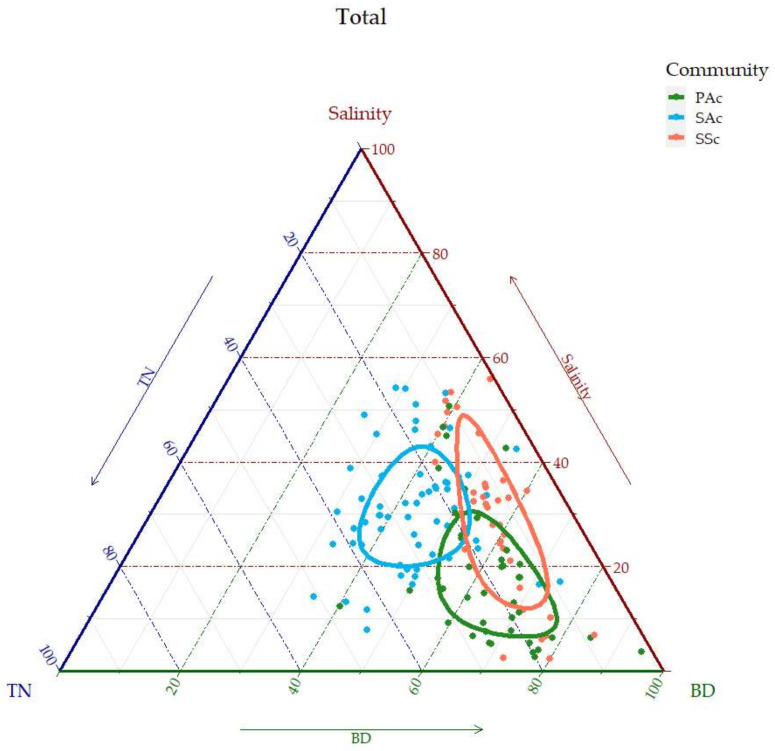
Ternary plots of community distribution. Different colors represent different communities. The three soil factor data were normalized, and the sum of the three equaled to one. Circles indicate 95% confidence intervals.

It can be seen that SAc generally occupied the area of higher TN and salinity (Figure 5). In contrast, SSc occupied the area of high salinity and high BD, and PAc occupied the area of high TN.

**Figure A2:**
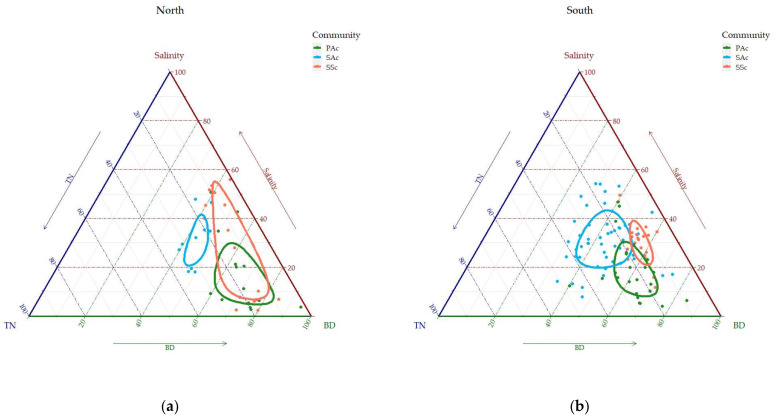

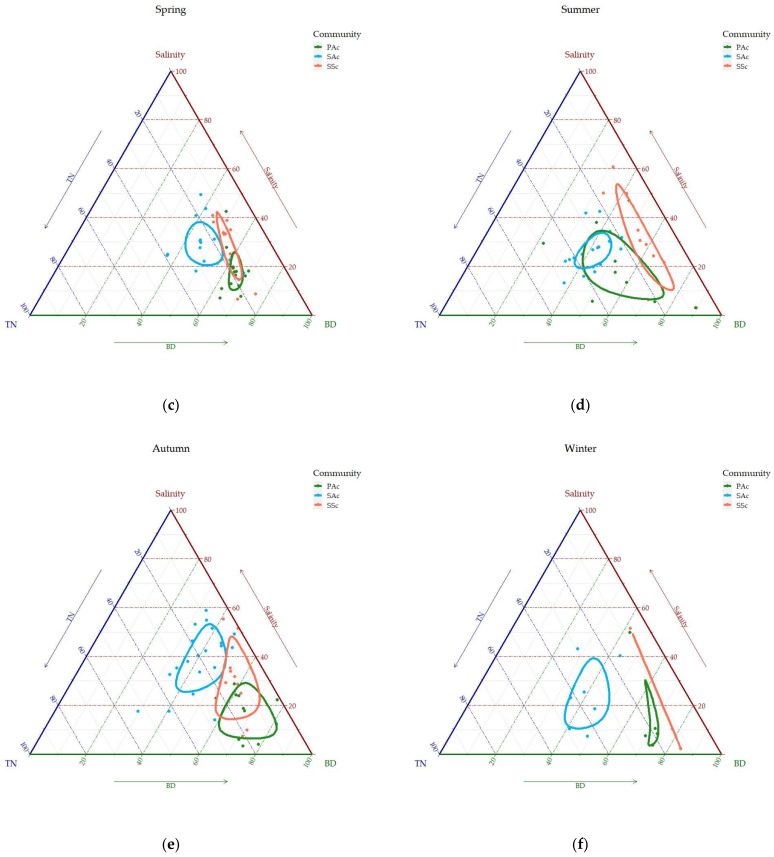
Ternary plots of community distribution in different regions and seasons: (**a**) North; (**b**) South; (**c**) spring; (**d**) summer; (**e**) autumn; and (**f**) winter. Different colors represent different communities. The three soil factor data were normalized and the sum of the three equaled to one. Circles indicate 95% confidence intervals.

Nevertheless, in the north and south and the four seasons, the degree of separation and the variation of distribution of the three communities were different (Figure A2). The three communities separated more in the south than in the north. Seasonally, SAc is distributed less separately in summer, while SSc separates significantly. In the south and in autumn, the three communities were more widely distributed among the three factors.

Therefore, although environmental heterogeneity (such as precipitation, evaporation, and temperature) caused by different geographical locations or seasons results in differences in the distribution of the three communities on the ternary plots, the choice of C–S–R strategy by species can still be identified. According to the C–S–R model and the distribution of the three communities in the plots, we could roughly recognize SA, SS and PA as C–S, S–R and C strategic species.

## 4. Discussion

### 4.1. Regional and Seasonal Differences of Soil Factors for Typical Wetland Communities

The study area along the coastal wetlands of YBS usually consisted of SAc, SSc and PAc, and there have been a number of studies about their corresponding soil factors, suggesting that in many cases, the BD of SSc is higher than that of SAc and PAc [18], TN of SAc is usually higher than that of PAc and SSc [19], and the salinity of PAc is lower than that of SAc and SSc [20]. However, in the process of data acquisition, it was found that the relative variation of factors was different in various sampling sites and seasons. For example, the BD of SSc was 1.17 ± 0.09 g/cm^3^ [21], which was lower than that of SAc (1.39 ± 0.09 g/cm^3^) [19] in autumn in different tidal flats by the Yellow Sea. The salinity of PAc was 0.68 ± 0.149% in spring [22], which was higher than that of SAc (0.37 ± 0.07%) in summer [23] at Yancheng coastal wetlands. The TN of SSc (0.517 ± 0.014 g/kg) at the Yellow River estuary in spring [24] was found to be higher than that of SAc (0.378 ± 0.108 g/kg) at the Wanggang coastal wetland in spring [19].

Through comprehensive analysis, we found that there is generally a difference in the relative variation of the main soil factors corresponding to communities. The soil factors of which there were the most significant differences among the three communities in the adaptive range were BD, TN and TP. Among them, in the north and south, the difference of BD was greater. Seasonally, in spring and autumn, the difference of BD and TP was more significant, and in the summer, the difference of soil TP was greater. In winter, the difference of soil BD and TN was greater.

Therefore, it is hard to reveal the differences of soil factors regardless of site location and season. To recognize the differences of soil factors corresponding to the communities, it is necessary to conduct a comprehensive comparison over different seasons and compare them as a whole. At the same time, the factors showing differences of communities among regions are different, so the differences of soil factors should be considered and the roles of each factor on vegetation differentiation should be analyzed according to the study area.

### 4.2. Driving Factors of Vegetation Differentiation

There exists a corresponding relationship between salt marsh vegetation and soil factors. Differences in soil factors among communities should represent a certain mechanism of vegetation differentiation. There were great differences in the explanation of vegetation differentiation in each region and season. Relevant studies are also controversial. Some studies in southeastern USA salt marshes suggested that salt marsh vegetation zonation may be caused by flooding and soil water potential [25], especially for the plants near sea level, which were more significant under flooding stress [26]. Meanwhile, among studies in the North Norfolk coast, UK, the Yellow River estuary, China, and the Chongming Island in the Yangtze River estuary, China suggested that elevation, pH and redox potential significantly affected the vegetation distribution pattern [27,28,29]. Some studies have shown that nutritional conditions cause zonation. The accumulation of nitrogen might be a significant factor leading to the succession of salt marsh vegetation [30]. Therefore, the roles of soil factors on vegetation differentiation are closely related to the study sites.

In this study area, the explained proportion of BD is the highest, followed by TN and salinity. TP and pH are relatively small, but the explained proportion of each factor is also different by season and region. In the north, the explained proportion of BD is dramatically high, up to 40%, while the explained proportion of other factors is relatively low. In the south, BD explained the highest proportion, but lower than that in the north, and TN explained the second highest proportion. In spring, the explained proportion of soil BD and TP is higher, in summer and winter soil BD and TN explained more proportion, and in autumn, the explained proportion of BD and salinity is higher.

The results are different from the existing studies. Although salinity and TN played an important role, BD was affected more. In general, the explanation of factors to vegetation differentiation is different by season and region, and the relative role is also different. Even in the same study area, the role of each driving factor was different, owing to the different seasons.

### 4.3. Plants’ Ecological Strategy and the Mechanism of Vegetation Differentiation

The three communities SAc, SSc, and PAc mostly exist as monodominant communities in tidal flats of the study area. In this specific habitat, the plants’ ecological strategies should have a certain corresponding relationship with the succession direction of the community. The soil factors impacting community distribution are also related to the choice of ecological strategies. Therefore, exploring ecological strategies and habitat requirements could explain the mechanism of vegetation differentiation to a certain extent. In this study, it was found that the three driving factors BD, salinity and TN are highly consistent with the community distribution.

Salinity is commonly recognized as a significant stress factor in tidal flats. Some studies have found that differences in salinity might be a determinant factor in plant species composition and distribution [31].

The utilization of nutrients by plants represented competitive ability [32]. In the nitrogen-limited salt marsh community, the competitive substitution of species mainly occurred in the competition for nutrients or the preferential occupation of spatial habitat [33]. Available nitrogen would facilitate the competitors [34].

BD plays an important role in vegetation differentiation and is positively related to perturbation in coastal wetlands. Bare lands and secondary bare lands were found in the tidal flats of the study area. These places are not covered with vegetation, and the soil is relatively compact, with high soil BD. They are mainly distributed in the middle tidal flat with high flooding frequency. The salinity changes considerably and the alternation of dry and wet is frequent, which is not suitable for vegetation colonization. As an annual herb, SS is a typical constructive species on the coastal beach [35], which is easy to plant on bare land or secondary bare land.

Combined with Grime’s C–S–R model [17,36,37], this study found that SA, SS and PA are C–S, S–R and C strategy species, respectively. From this study, it can be seen that there is a corresponding relationship between species’ ecological strategies and communities’ habitat characteristics, and further, the driving forces of vegetation differentiation. Based on the corresponding relationship between the selection of ecological strategies and vegetation differentiation, species’ ecological strategies can be considered in the restoration of the coastal wetland ecosystem. Creating the corresponding habitats might benefit the specific species.

Along the coastal wetlands of YBS, with the invasion of SA and change in the hydrological process, the SSc was significantly degraded [36]. Due to the transformation of the hydrological process by SAc, PAc also occupied part of the niche of the SSc [37,38]. To restore the native vegetation, it is of significant importance to recognize the driving forces on vegetation differentiation and the ecological strategies chosen by plant species. There might be an effective way to control SAc and PAc and restore SSc by creating a moderate perturbation environment and continuously forming secondary bare lands. Additionally, vegetation restoration and management could be carried out considering the ecological strategy selection by plants to achieve the purpose of plant diversity conservation.

## 5. Conclusions

Through the analysis of the published data, we found that the differences of the main soil factors corresponding to the typical communities in the study area were mostly represented by BD, salinity and TN. The differences in factor variation range among communities changed with site locations and seasons, and different factors also showed different variation characteristics in each community type.

Through the corresponding analysis, we found that BD, salinity and TN played a comparably more critical role on vegetation differentiation in tidal flat wetlands. Generally, SAc was positively related to TN, SSc was positively related to BD and salinity, and PAc was negatively related to salinity.

Cosidering BD was positively related to the frequency of perturbation, salinity represents the environmental stresses, and higher TN facilitating the competitors in the specific habitat of the tidal flats by YBS, SA, SS and PA could be recognized as C–S, S–R and C strategic species according to Grime’s C–S–R model to some extent.

The results of this study could be applied for the management and restoration of vegetation and coastal wetland ecosystems by the YBS.

## Figures and Tables

**Table 1:** Overlap of soil factor variation ranges of different communities by region and season.

Soil Factors		Total		Regions				Seasons							
				North		South		Spring		Summer		Autumn		Winter	
		SSc	PAc	SSc	PAc	SSc	PAc	SSc	PAc	SSc	PAc	SSc	PAc	SSc	PAc
BD	SAc	0.34	0.54	0.01	0.01	0.09	0.10	0.09	0.14	0.11	0.29	0.18	0.13	0.01	0.04
	SSc	−	0.55	−	0.75	−	0.08	−	0.18	−	0.25	−	0.21	−	0.26
Salinity	SAc	0.77	0.41	0.39	0.40	0.68	0.30	0.66	0.24	0.32	0.33	0.29	0.22	0.46	0.11
	SSc	−	0.42	−	0.36	−	0.30	−	0.25	−	0.50	−	0.38	−	0.12
pH	SAc	0.74	0.73	0.57	0.64	0.57	0.55	0.32	0.27	0.52	0.75	0.44	0.47	0.43	0.43
	SSc	−	0.86	−	0.77	−	0.72	−	0.52	−	0.59	−	0.72	−	0.85
TN	SAc	0.34	0.42	0.23	0.42	0.24	0.33	0.30	0.27	0.18	0.56	0.35	0.37	0.17	0.01
	SSc	−	0.70	−	0.34	−	0.42	−	0.61	−	0.28	−	0.54	−	0.05
TP	SAc	0.47	0.50	0.38	0.52	0.49	0.16	0.08	0.43	0.02	0.38	0.25	0.18	0.25	0.17
	SSc	−	0.47	−	0.63	−	0.25	−	0.08	−	0.03	−	0.17	−	0.23

**Table 2:** Statistical results based on ANOVA of communities and environmental factors in RDA.

Variables	R^2^	Adjusted R^2^	F	*p* Values
Total	0.380331	0.358199		
BD			39.8	0.002 **
Salinity			17.5	0.002 **
pH			15.3	0.002 **
TN			1.3	0.26
TP			1	0.342

Note: Asterisks represent significance, where ‘**’ represents *p* < 0.01.

**Table 3:** Eigenvalues and correlation coefficients of communities and environmental factors on the first two axes of RDA.

Statistic	Eigenvalues	Explained Variation (Cumulative)	Pseudo-Canonical Correlation	Explained Fitted Variation (Cumulative)
Axis 1	0.317	31.66	0.753	83.25
Axis 2	0.064	38.03	0.380	100

**Table 4:** Simple effect of soil factors among different regions and seasons.

Ranking	Total	Regions		Seasons			
		North	South	Spring	Summer	Autumn	Winter
1	BD (21.7% **)	BD (34.3% **)	BD (15.3% **)	BD (23.2% **)	BD (26.2% **)	BD (35.3% **)	TN (43.4% **)
2	TN (15.4% **)	TN (6.4% †)	TN (16.4% **)	TP (21.0% **)	TN (16.4% **)	Salinity (15.8% **)	BD (28.3% **)
3	Salinity (7.1% **)	Salinity (7.8% *)	Salinity (11.6% **)	TN (18.1% **)	TP (8.2% **)	TN (11.6% **)	pH (18.7% †)
4	TP (2.6% *)	pH (3.9%)	TP (1.6%)	Salinity (12.4% **)	Salinity (4.9% †)	TP (6.5% †)	TP (4.3%)
5	pH (0.4%)	TP (2.4%)	pH (0.7%)	pH (0.2%)	pH (<0.1%)	pH (0.3%)	Salinity (1.5%)

Note: Asterisks represent significance, where ‘**’ represents *p* < 0.01, ‘*’ represents *p* < 0.05, and ‘†’ represents *p* < 0.1.

## Data Availability

The data presented in this study are available on request from the corresponding author. The soil factors data underlying this study are available at https://github.com/ecodq/vegetation−differentiation.git (accessed on 5 November 2021). Citations for the soil factors data are provided in the methods.

## Appendix A

**Table A1:** Citation information.

Article	Commuities	Location	Season	Environmental Factors	Citation
Changes in soil microbial biomass and community composition in coastal wetlands affected by restoration projects in a Chinese delta	SS, PA	North	Spring	pH, Salinity	[39]
Characterization of the salt marsh soils and visible−near−infrared spectroscopy along a chronosequence of *Spartina alterniflora* invasion in a coastal wetland of eastern China	SA	South	Autumn	BD, pH, Salnility, TN	[40]
Comparison of phosphorus fractions and phosphatase activities in coastal wetland soils along vegetation zones of Yancheng National Nature Reserve, China	SA, SS, PA	South	Summer	pH, Salinity, TN, TP	[41]
Consequences of short−term C−4 plant *Spartina alterniflora* invasions for soil organic carbon dynamics in a coastal wetland of Eastern China	SA, SS, PA	South	Spring	BD, pH, Salnility	[20]
Decomposition processes in coastal wetlands: the importance of *Suaeda salsa* community for soil cellulose decomposition	SS, PA	North	Autumn	Salinity, TN, TP	[42]
The effect of biomass variations of *Spartina alterniflora* on the organic carbon content and composition of a salt marsh in northern Jiangsu Province, China	SA	South	Spring, Summer, Autumn, Winter	TN	[43]
Effects of invasion of *Spartina alterniflora* and exogenous N deposition on N_2_O emissions in a coastal salt marsh	SA, PA	South	Spring	BD, TN	[19]
Effects of *Spartina alterniflora* invasion and exogenous nitrogen on soil nitrogen mineralization in the coastal salt marshes	SA, SS, PA	South	Summer	BD, Ph, TN	[44]
Effects of *Spartina alterniflora* Invasion on Soil Respiration in the Yangtze River Estuary, China	SA, PA	South	Autumn	pH	[45]
Exotic *Spartina alterniflora* provides compatible habitats for native estuarine crab Sesarma dehaani in the Yangtze River estuary	SA, PA	South	Summer	pH, Salinity	[46]
Halophyte Plant Communities Affecting Enzyme Activity and Microbes in Saline Soils of the Yellow River Delta in China	SS, PA	North	Spring, Summer, Autumn	Salinity	[11]
The impact of sea embankment reclamation on soil organic carbon and nitrogen pools in invasive *Spartina alterniflora* and native *Suaeda salsa* salt marshes in eastern China	SA, SS	South	Autumn	BD, PH, Salinity	[21]
Impacts of Age and Expansion Direction of Invasive *Spartina alterniflora* on Soil Organic Carbon Dynamics in Coastal Salt Marshes Along Eastern China	SA, SS	North	Autumn	BD, pH, Salinity, TN	[18]
Impacts of burial by sediment on decomposition and heavy metal concentrations of *Suaeda salsa* in intertidal zone of the Yellow River estuary, China	SS	North	Spring	pH, Salinity, TN	[47]
Impacts of *Spartina alterniflora* invasion on soil organic carbon and nitrogen pools sizes, stability, and turnover in a coastal salt marsh of eastern China	SA, SS, PA	South	Autumn	BD, PH, Salinity	[48]
Plant litter composition selects different soil microbial structures and in turn drives different litter decomposition pattern and soil carbon sequestration capability	SA, PA	South	Autumn	pH, TN	[49]
Response of methane emission to invasion of *Spartina alterniflora* and exogenous N deposition in the coastal salt marsh	SA, SS	South	Spring	BD, TN	[50]
Seasonal Dynamics of Trace Elements in Tidal Salt Marsh Soils as Affected by the Flow−Sediment Regulation Regime	SS, PA	North	Spring, Summer, Autumn	BD, Salinity, pH	[12]
Short−term C−4 plant *Spartina alterniflora* invasions change the soil carbon in C−3 plant−dominated tidal wetlands on a growing estuarine Island	SA	South	Autumn	TN	[51]
Soil fungal communities vary with invasion by the exotic Spartina alternifolia Loisel. in coastal salt marshes of eastern China	SA, SS, PA	South	Winter	pH, Salinity	[52]
Soil organic carbon of degraded wetlands treated with freshwater in the Yellow River Delta, China	SS, PA	North	Spring	PH, Salinity, TN	[24]
Two−decade wetland cultivation and its effects on soil properties in salt marshes in the Yellow River Delta, China	SS, PA	North	Winter	BD, Salinity, pH, TN, TP	[53]
Analysis on Diversity of Soil Bacterial Community in Original Coastal Wetland of Yancheng, Jiangsu	SA, SS, PA	South	Autumn	pH, TN, TP	[54]
The Assessment of Carbon Storage of Vegetation Zones in the Jiuduan Shoal Wetland	SA, PA	South	Spring, Summer, Autumn, Winter	BD	[55]
Biologically−Based Availability and Influencing Factors of Soil Phosphorus under Different Vegetation in Coastal Beach Wetlands	SA, PA	South	Spring	pH, TN, TP	[56]
Carbon, nitrogen and phosphorus content and ecological stoichiometry of *Spartina alterniflora* in the tidal flat wetland of Jiaozhou Bay	SA	North	Spring, Summer, Autumn, Winter	BD, Salinity, pH, TN, TP	[57]
Characteristics and Factors of Soil Enzyme Activity for Different Plant Communities in Yellow River Delta	SS, PA	North	Summer	Salinity, pH, TN, TP	[58]
The characteristics and mechanism of landscape evolution in the coastal wetlands under natural and human influence	SA, SS, PA	South	Spring	Salinity	[59]
Characteristics of halophyte and associated soil along aggradational muddycoasts in Jiangsu Province	SA, SS	South	Spring	Salinity, TN, TP	[60]
The Characteristics of Surficial Sediments Organic Carbon inYancheng Coastal Wetland	SA, SS, PA	South	Spring	BD, Salinity, pH	[61]
Contents of Organic Carbon and Dissolved Organic Carbon and Characteristics of Functional Group Structure in Surface Soils of Salt Marshes in Yellow River Delta	SA, SS, PA	North	Summer	pH, Salinity	[62]
The Coupling Relationship between Soil Eco−Processes and Landscape Evolution under the Natural Conditions in Yancheng Coastal Wetland	SA, SS, PA	South	Spring	Salinity	[63]
Distribution and Influence factors of soil organic carbon of different land−use types in the Jiangsu coastal areas	SA, SS	South	Autumn	pH, TN, TP	[64]
Distribution characteristic and spatial heterogeneity of soil organic carbon on the south coastal of Hangzhou Bay	SA, PA	South	Spring	pH, Salinity, TN	[65]
Distribution characteristics of organic carbon and its components in soils under different types of vegetation in wetland of Hangzhou Bay	SA, PA	South	Spring	pH, Salinity	[66]
Distribution Characteristics of Phosphorus under Different Vegetation Communities in Salt Marshes of Jiaozhou Bay Communities in Salt Marshes of Jiaozhou Bay	SA, SS, PA	North	Autumn	TP	[67]
Diversity survey in rhizosphere of diazotroph in the exotic invasive species *Spartina alterniflora* and two native species (*Phragmites australis* and Scripus mariqueter) in the wetlands at Chongming Dongtan in the Yangtze River estuary	SA, PA	South	Spring	pH	[68]
Ecological mechanisms of vegetation succession of coastal wetland in Yancheng Nature Reserve	SA, SS, PA	South	Spring	Salinity	[22]
Effect of litter decomposition on mineralization of soil organic carbon in the Jiaozhou Bay coastal wetlands	SA, SS, PA	North	Winter	pH, Salinity, TN, TP	[69]
Effect of Salt on Soil Nitrogen Mineralization in Coastal Wetland of Liaohe Estuary	SS, PA	North	Spring	pH, TN	[70]
Effect of *Spartina alterniflora* Invasion on Coastal Wetland Soil Carbon Pool and Stability in Subtropical China	SA, PA	South	Summer	BD, pH, Salinity, TN, TP	[71]
Effects of plant invasion along a Spartina alterniflora chronosequence on organic carbon dynamics in coastal wetland in north Jiangsu.	SA, SS	South	Autumn	BD	[72]
Effects of plant invasion on soil caibon dynamics and CH4 emissions from coastal wetlands	SA, PA	South	Summer	TN	[73]
The effects of salt marsh vegetation on soil organic carbon fractions, sources and distribution	SA, SS	South	Summer	BD, pH, Salinity, TN	[74]
Effects of *Spartina alterniflora* Invasion on Soil Carbon Fractions in Mangrove Wetlands of China	SA	South	Summer	BD, pH, Salinity, TN, TP	[75]
Effects of *Spartina alterniflora* invasion in eastern Fujian coastal wetland on the physicochemical properties and enzyme activities of mangrove soil.	SA	South	Autumn	Ph, TN, TP	[76]
The Key Factor of Impact on Temporal and Spatial Variation of Soil Organic Matter, TN and TP in Coastal Salt Marsh: Tide and Vegetation	SA, SS	South	Spring, Summer, Autumn	TN, TP	[77]
Leaching Characteristics of Soil Dissovled Organic Carbon in Coastal Wetlands of Jiaozhou Bay	SA, SS, PA	North	Summer	BD, pH, Salinity	[78]
Morphology and Biomass Distribution of *Spartina alterniflora* Growing in Different Tidal Flat Habitats in Jiangsu	SA	South	Autumn	pH, Salinity, TN, TP	[79]
Nutrient dynamics of litter−soil system during litter decomposition in coastal wetlands of Jiaozhou Bay	SA, SS, PA	North	Autumn	Ph, TN, TP	[80]
Relative competitive ability of *Spartina alterniflora* patches to native species in tidal zone ecotone of north Jiangsu	SA, SS	South	Summer	pH, Salinity	[81]
The relative importance and mechanism of soil dissimilatory nitrate reduction to ammonium and denitrification under the change of land use: A case study in chongming dongtan	SA, PA	South	Spring, Summer, Autumn, Winter	BD, pH, Salinity	[82]
Research on characteristics of vegetation distribution pattern and soil factors in the intertidal zone of Zhimai River estuary	SA, PA	North	Summer	pH	[83]
Respirations and Response in Temperature of Salt Marsh Soil in Different Types of Wetlands Along the Coast of Yancheng	SA, PA, SS	South	Spring	pH, Salinity, TN	[84]
The response of organic carbon content to biomass dynamics in *Spartina alterniflora* marsh.	SA	South	Spring, Summer, Autumn, Winter	TN	[85]
Retention Effect of Wetland for Nitrogen and Phosphorus Nutrients in the Coastal Zone of the Yancheng	SA	South	Summer	TN, TP	[86]
Soil Quality Evaluation of Bare Flat and Salt Marshes in Jiaozhou Bay Wetlands	SA, SS, PA	North	Summer	BD, pH, Salinity, TN, TP	[87]
Spatial Distribution and Influencing Factors of the Biomass of Spartina alterniflora in Coastal Wetlands of Zhejiang	SA	South	Summer	pH, TN, TP	[88]
Spatial Heterogeneity of Soil Salinity in Jiangsu Yancheng Wetland National Nature Reserve Rare Birds	SA, SS, PA	South	Spring	Salinity	[89]
The stoichiometric characteristics of different plant communities in the Duliujian River estuary	SA, SS, PA	North	Autumn	pH, Salinity	[90]
Study on CH4 Emission Fluxes in Hangzhou Bay Coastal Wetland	SA, PA	South	Autumn	PH, Salinity, TN	[91]
Study on methane, nitrous oxide and carbon dioxide fluxes and their influencing factors in Hangzhou Bay coastal wetland	SA, PA	South	Autumn	PH, Salinity, TN	[92]
Temporaland Spatial Variability of Soil Nutrients in Different Vegetation Zones of Yueqing Bay Coastal Wetlands	SA	South	Summer, Winter	TN, TP	[93]
Vertical distribution and seasonal variation of nitrogen, phosphorus elements in *Spartina alterniflora* wetland of Jiaozhou Bay, Shandong, China	SA	North	Spring, Summer, Autumn	TN, TP	[94]

**Table A2:** Statistical results based on ANOVA of communities and environmental factors in RDA.

Variables	R^2^	Adjusted R^2^	F	*p* Values
North	0.458549	0.389132		
BD			22.4	0.002 **
Salinity			5.7	0.032 *
pH			1.2	0.292
TN			1	0.332
TP			0.5	0.556
South	0.37053	0.3374		
BD			19.4	0.002 **
Salinity			14.2	0.002 **
pH			13.6	0.002 **
TN			0.9	0.394
TP			0.8	0.464
Spring	0.554406	0.498707		
BD			13.3	0.002 **
Salinity			8.8	0.002 **
pH			7.4	0.004 **
TN			8.7	0.002 **
TP			0.1	0.86
Summer	0.334422	0.253254		
BD			15.9	0.002 **
Salinity			2.7	0.058 †
pH			1.7	unknown
TN			0.1	0.852
TP			0.1	0.852
Autumn	0.504579	0.43577		
BD			21.8	0.002 **
Salinity			10.2	0.002 **
pH			0.8	0.382
TN			0.5	0.594
TP			<0.1	0.982
Winter	0.731209	0.58188		
BD			10	0.002 **
Salinity			6.2	0.014 *
pH			2	0.184
TN			1.1	unknown
TP			0.5	0.576

Note: Asterisks represent significance, where ‘**’ represents *p* < 0.01, ‘*’ represents *p* < 0.05, and ‘†’ represents *p* < 0.1.

**Table A3:** Eigen values and correlation coefficients of communities and environmental factors on the first two axes of RDA.

Statistic		Eigenvalues	Explained Variation (Cumulative)	Pseudo-Canonical Correlation	Explained Fitted Variation (Cumulative)
North	Axis 1	0.360	35.99	0.922	78.49
	Axis 2	0.099	45.85	0.414	100
South	Axis 1	0.278	27.76	0.685	74.93
	Axis 2	0.093	37.05	0.477	100
Spring	Axis 1	0.388	38.82	0.891	70.01
	Axis 2	0.166	55.44	0.571	100
Summer	Axis 1	0.304	30.35	0.754	90.77
	Axis 2	0.031	33.44	0.257	100
Autumn	Axis 1	0.477	47.72	0.908	94.58
	Axis 2	0.027	50.46	0.255	100
Winter	Axis 1	0.499	49.87	0.906	68.2
	Axis 2	0.233	73.12	0.769	100
